# A Case of Diphtheria Treated with Antitoxin*The above is the article from the *Medical News* referred to by Dr. Preston in the preceding letter entitled “Waiting upon the Remedy.”

**Published:** 1894-12

**Authors:** Arnold W. Catlin

**Affiliations:** Brooklyn, N. Y.


### A CASE OF DIPHTHERIA TREATED WITH THE ANTITOXIN.*

ARNOLD W. CATLIN, M. D., BROOKLYN, N. Y.

Arthur H., aged nine years, first complained of bring unwell Saturday, October 6th. There was general malaise; the child was feverish and debilitated; there was loss of appetite, and he was disposed to maintain a recumbent posture. He was first seen on Sunday morning, October 7th. Debility was extreme, the skin hot and dry; the temperature 102°, the pulse 120. The tongue was coated and the breath very foul. Examination of the throat showed a mass of exudation completely filling the fauces, and nasal breathing was obstructed. Diphtheria of a low type was diagnosticated. He was put upon the Mercuric chloride, gr. 1/40, and tr. ferri chlor., ♏v, every three hours, and the throat was sprayed hourly with a weak solution of Mercuric chloride. He was nourished with whisky punch freely, and a culture-tube was immediately forwarded to the Board of Health.

I secured five grams of the antitoxin (Aronson's *Heilserum*), and gave the whole amount in one hypodermic injection between the shoulder-blades, according to the method used in Berlin, and explained by Dr. Louis Fischer, of New York. This was at 7 P. M. Sunday, when the disease had been present certainly forty-eight hours, and probably, in view of the fact

---

* The above is the article from the *Medical News* referred to by Dr. Preston in the preceding letter entitled "Waiting upon the Remedy."

that so much surface was involved by the exudate, for seventy-two hours, although no attention was called to the throat by the patient until the morning of the day the antitoxin was used.

No reaction followed the hypodermic injection. The patient passed a restless night, and was found no better on Monday morning. The report from the Board of Health as to the culture was: "True diphtheria." The cadaveric odor from the throat was almost intolerable. The heart's action was weak, and debility was even more pronounced than on the previous day. The temperature was from 100.5° to 102°; the pulse was 120 and feeble. The child positively refused stimulants in any form by the mouth. The condition of the throat made deglutition difficult. The appearance of the exudate was about the same as at the time of the injection the night before. Continuance of treatment was directed, with the constant use of hot brandy to the surface, and hot brandied cloths to the abdomen continuously. On Monday evening no progress was noted, and the case was assuming a grave aspect. The prognosis was unfavorable, and the new remedy was considered *inert.* On Tuesday morning, thirty-six hours after the use of the antitoxin, there was an evident change for the better. The temperature had fallen, the pulse was stronger, the tongue cleaner, and the exudate was loosening at the edges, and the surrounding parts looked bright and healthy. The improvement continued throughout the day. There was a free flow of saliva and mucus from the throat, but no membrane exfoliated. It appeared thinner, and was evidently disappearing.

The child was more comfortable that night, and evidences of gain in every way were noted on Wednesday morning. The temperature was nearly normal, the pulse 86, the odor from the throat nearly gone, and the membrane was rapidly disappearing. The underlying surface could be seen, and the whole appearance was fresh and healthy. No masses or flakes of membrane were loosened or coughed up. It all appeared to be melting away. On Thursday the temperature touched normal, the pulse was 86, the throat was nearly clear, and the nasal passages were much freer. The patient declared himself well, and his general tone was certainly excellent. He was hungry, and called for solid food. His urine was examined and found free from albumin. On Friday all the visible part of the throat was free from exudate, the tongue was slightly coated, but moist at the edges, the breath was entirely free from odor, the temperature ranged from 97.6° to 99°.

The report on the cultures, taken Thursday, was that the "Klebs-Lœffler bacilli were still present." On Saturday the child was, to all appearance, well; the throat was normal, and the nasal passages clear. All evidence of the disease had disappeared; and the temperature averaged normal. He had a royal appetite. On Sunday, one week from the date of the use of the antitoxin, the child would have been declared quite well if the report on the culture had not read "bacilli still present." I kept the child in bed and quarantined for the ensuing week, and on the eleventh day sent cultures to the laboratories of both the New York and Brooklyn Boards of Health to secure comparative tests. Reports from both read, "bacilli present." Persistent nutrition was kept up, and the throat sprayed with Seiler's solution and Mercuric chloride alternately.

Cultures made on the seventeenth day showed bacilli still present, but I had been unable to detect the slightest evidence of the disease since Saturday, October 13th, six days after the use of the antitoxin. Unfavorable reports from the Board of Health continued until the twenty-first day, when the cultures were declared free of bacilli. An intercurrent attack of follicular tonsillitis, however, again showed them present until the twenty-ninth day, when they had permanently disappeared.

No albumin was found during the course of the disease save on the eighth day, and then only a trace.

This case has been of great value as showing: first, the wonderful abortive power of the new remedy in dealing with this treacherous disease; and, second, as demonstrating its ability to limit the absorption of the diphtheric poison and so prevent the sequelæ so often seen and so fatal in a certain proportion of those recovering from the acute or primary stage of the disease. I refer especially to the cardiac and renal complications.

The presence of the true diphtheric bacilli for twenty-nine days in cultures made from the throat, three weeks after all distinctive symptoms had disappeared, and the child was apparently well, is very suggestive.

It certainly emphasizes the wisdom of making cultures and the need of quarantining patients as long as an unfavorable report comes back from the Board of Health. Doubtless this is one explanation of the spread of this direful disease in our great cities, where the children herd together in our ward schools and infect each other during the period of convalescence, when, if cultures had been made, they would still be in quarantine and the mischief prevented.—*Medical News.*

